# Outcome of Transplant Recipients Infected with Omicron BA.1 and BA.2: A Single-Center Retrospective Study in Saudi Arabia

**DOI:** 10.1007/s44197-023-00084-6

**Published:** 2023-01-10

**Authors:** Abeer N. Alshukairi, Yasser Aldabbagh, Sabir A. Adroub, Tobias Mourier, Khalid Y. Abumelha, Ghadeer E. Albishi, Basem M. Alraddadi, Mohammad K. Al Hroub, Aiman El-Saed, Suzan M. Nagash Ibrahim, Mohammed Al Musawa, Ahlam Almasari, Wael T. Habahab, Fatimah S. Alhamlan, Awad Al-Omari, Arnab Pain, Ashraf Dada

**Affiliations:** 1 Department of Medicine, King Faisal Specialist Hospital and Research Center, King Faisal Specialist Hospital and Research Center, PO BOX 40047, Jeddah, 21499 Kingdom of Saudi Arabia; 2 College of Medicine, AlFaisal University, Riyadh, Kingdom of Saudi Arabia; 3 Department of Medicine, Al-Moosa Specialist Hospital, Al-Ahsa, Kingdom of Saudi Arabia; 4 Pathogen Genomics Group, Bioscience Program, BESE Division, King Abdullah University of Science and Technology (KAUST), Thuwal, Kingdom of Saudi Arabia; 5 Department of Pathology and Laboratory Medicine, King Faisal Specialist Hospital and Research Center, Jeddah, Kingdom of Saudi Arabia; 6 Department of Infection Control and Hospital Epidemiology, King Faisal Specialist Hospital and Research Center, Jeddah, Kingdom of Saudi Arabia; 7 Department of Infection Prevention and Control, King Abdulaziz Medical City, Riyadh, Kingdom of Saudi Arabia; 8 Department of Oncology, King Faisal Specialist Hospital and Research Center, Jeddah, Kingdom of Saudi Arabia; 9 Department of Medical and Critical Care Pharmacy, King Faisal Specialist Hospital and Research Center, Jeddah, Kingdom of Saudi Arabia; 10 Department of Infection and Immunity, King Faisal Specialist Hospital and Research Center, Riyadh, Kingdom of Saudi Arabia; 11 Department of Critical Care, Dr Sulaiman Al Habib Medical Group, Riyadh, Kingdom of Saudi Arabia

**Keywords:** COVID-19, Outcome, Omicron variant, Transplantation

## Abstract

The outcome of transplant recipients is variable depending on the study population, vaccination status and COVID-19 variants. Our aim was to study the impact of Omicron subvariants on the mortality of transplant recipients. We reviewed the results of SARS-CoV-2 whole genome sequence of random isolates collected from 29 December 2021 until 17 May 2022 in King Faisal Specialist Hospital and Research center, Jeddah (KFSHRC-J), Saudi Arabia performed as hospital genomic surveillance program for COVID-19 variants. We included 25 transplant patients infected with confirmed Omicron variants.17 (68%) and 8 (32%) patients had Omicron BA.1 and BA.2, respectively. 12 (68%) patients had renal transplants. Only 36% of patients received three doses of COVID-19 vaccines. 23 (92%) patients required hospitalization. 20 (80%) patients survived and 6 (25%) required intensive care unit (ICU) admission. Among ICU patients, 66.7% were more than 50 years, 50% had two to three comorbidities and 5 out of 6 (83%) died. The mortality of transplant patients infected with Omicron variants in our cohort was higher than other centers as a limited number of patients received booster vaccines. Optimizing booster vaccination is the most efficient method to improve the mortality of COVID-19 in transplant recipients recognizing the inefficacy of monoclonal antibodies in the presence of SARS-CoV-2 emerging variants. We did not show a difference in mortality in transplant patients infected with Omicron BA.1 and BA.2 knowing the limitation of our sample size.

## Introduction

The outcome of transplant patients infected with SARS-CoV-2 is variable in different studies; while some studies showed that their mortality is higher than the general population, others demonstrated that the outcome of COVID-19 in transplant patients depends on the age and the associated comorbidities [1]. Although the COVID-19 vaccine immune responses were suboptimal in preventing breakthrough infections in transplant recipients during the Delta and Omicron surge, they were effective in reducing their mortalities in a large multicenter study in USA [2]. A recent national Danish study showed a lower mortality rate among solid organ transplant recipients compared to the general population during the omicron surge versus the pre-omicron period [3]. We described the outcome of a cohort of transplant recipients infected with Omicron variants in a tertiary care center in Jeddah, Saudi Arabia and we observed the impact of Omicron subvariants on mortality.

## Methods

### Study Population

Since the emergence of SARS-CoV-2 variants, samples of confirmed COVID-19 cases were stored in the virology laboratory, in KFSHRC-J. These samples were included in the hospital genomic surveillance program for COVID-19 variants. They were transported to King Abdullah University of Science and Technology (KAUST), Thuwal, and King Faisal Specialist Hospital and Research Center, Riyadh (KFSHRC-R), in Saudi Arabia, every three to six months for whole genome sequence analysis We reviewed the results of SARS-CoV-2 whole genome sequence analysis of isolates collected from 29 December 2021 until 17 May 2022 in KFSHRC-J. During this time period, Omicron was the predominant circulating SARS-CoV-2 variant. We included solid organ and hematopoietic transplant patients with confirmed Omicron variants BA1 and BA2 that were home isolated and hospitalized. We excluded non-transplant recipients and patients with non-Omicron variants. We also excluded pediatric patients (less than 14 years according to the hospital definition) and patients with unidentified variants.

### Data Collection

Electronic medical records were retrospectively reviewed for age, gender, comorbidities, disease severity, ICU admission, home isolation versus hospitalization, COVID-19-specific therapy, immunosuppressive medications, vaccination status (type and number of vaccine), time between last vaccine dose and date of confirmed COVID-19 diagnosis and outcome.

### The Whole Genome Sequence Analysis

The whole genome sequence analysis was performed in KAUST and KFSHRC-R in Saudi Arabia as previously described [4, 5].

### The Whole Genome Sequence Analysis in KFSHRC-R

The samples were subjected to nucleic acid extraction using a MagMAX^™^ Viral/Pathogen Nucleic Acid Isolation Kit (Cat No. A42352, Thermo Fisher Scientific; MA, USA). All positive samples for SARS-CoV-2 were converted to cDNA using SuperScript^™^ IV VILO^™^ Master Mix (ThermoFisher Scientific, USA). The cDNAs were amplified using the Ion AmpliSeq^™^ SARS-CoV-2 Insight Research Assay as indicated by the manufacturer’s instructions. Amplified products were ligated with unique barcode adaptors using the Ion Xpress Barcode Adaptors 1–16 kit (ThermoFisher Scientific, USA) and purified with 1.5 × volume of Agencourt AMPure XP Reagent (Beckman Coulter, USA). Library was built and normalized to 33 pM using nuclease-free water, and up to 16 libraries were equally pooled for further processing. Pooled libraries were used as a template input for emulsion PCR and enrichment of template-positive particles using the Ion Chef automated system with the Ion 510 Kit-Chef kit (ThermoFisher Scientific, USA) according to the manufacturer’s instructions. The obtained data were primarily processed (base calling, base quality recalibration, alignment, assembly, and variant calling) with Torrent Suite Server, version 5.12 (ThermoFisher Scientific, USA). De novo assembly of the contigs was performed using the assembly Trinity plugin (v1.2.1), and consensus sequences of each sample were generated using the IRMA plugin (v1.2.1). Variant call files were analyzed on the COVID19AnnotateSnpEff plugin to identify and annotate variants with public and private databases.

### The Whole Genome Sequence Analysis in KAUST

200 μl of patient’s nasopharyngeal swap stored in viral transport medium (VTM) was used for total RNA extraction using the RNAdvance viral RNA extraction kit from Beckman Coulter (https://www.beckman.com/reagents/genomic/rna-isolation/viral) according to the manufacturer instructions and specifications and with one modification of eluting the purified RNA in 100ul. To determine *C*_q_ values and to confirm the positive samples, we performed quantitative RT-PCR. We used the 4 × TaqPath^™^ 1-Step RT-qPCR Master Mix, CG (ThermoFisher, Cat number A15299), according to manufacturer specifications using 2.5 µl of RNA as an input template. Primers and probes were obtained from IDT (IDT, Cat number 10006606) targeting the N1 and N2 regions of the nucleocapsid phosphoprotein of SARS-CoV-2. NP targets the RNase P gene for detection of human nucleic acids and to act as control for sample quality and integrity. Samples were prepared for sequencing following the SARS-Cov2 genome sequencing protocol midnight using Oxford Nanopore Rapid barcoding kit (https://www.protocols.io/view/sars-cov2-genome-sequencing-protocol-1200bp-amplic-rm7vz8q64vx1/v6) with one modification of using artic primer set V3 and V4.1 (https://github.com/joshquick/artic-ncov2019/tree/master/primer_schemes/nCoV-2019/V3) to generate 400 bp amplicon instead of using midnight 1200 bp amplicon.

Oxford Nanopore Rapid Barcoding kit (https://store.nanoporetech.com/productDetail/?id=rapid-barcoding-kit-1) were used for barcoding samples for multiplexing and the barcoded samples were then loaded and sequenced on MinION MK1C platform. Generated data were analyzed using the wf-artic pipeline from Epi2me-labs (https://github.com/epi2me-labs/wf-artic) and the SARS-CoV-2 variants were called.

### COVID-19 Guidelines in KFSHRC-J

Patients with confirmed COVID-19 and admitted to KFSHRC-J were treated according to the hospital COVID-19 guidelines. These guidelines were developed according to the international COVID-19 treatment guidelines based on the review of clinical pharmacists and infectious diseases consultants. In brief, immunocompromised patients with COVID-19 upper respiratory tract infection, were hospitalized to receive 3 doses of daily remdesivir. Patients with COVID-19 pneumonia requiring oxygen supplementation were treated with 5 doses of daily remdesivir and 10 doses of daily dexamethasone. Tocilizumab is administered for COVID-19 patients requiring high flow oxygen and those on mechanical ventilation.

### Statistical Analysis

Demographic and clinical characteristics of the patients were compared by death status. Fisher exact test was used to examine significant differences in categorical variables, while Mann–Whitney was used to examine significant differences in continuous variables. SPSS (Version 25.0. Armonk, NY: IBM Corp) was used for all statistical analyses.

## Results

Whole genome sequence was performed on 268 random isolates of SARS-CoV-2. 25 transplant patients with confirmed omicron variant of COVID-19 were included in the current analysis. 17 (68%) patients had Omicron BA.1 and 8 (32%) patients had Omicron BA.2. 12 (68%) patients had renal transplant. 56% of patients were males and the mean age was 49 years. The most frequent comorbidity was diabetes mellitus (52%). The most frequent immunosuppressive medications received included tacrolimus (88%) and mycophenolate (80%). 48% and 36% of patients received two and three COVID-19 vaccine doses, respectively. Among 22 vaccinated transplant recipients, 19 (86.3%) and 3 (13.7%) of patients received Pfizer-BioNTech and Oxford/AstraZeneca COVID-19 vaccines. 23 (92%) patients required hospitalization for COVID-19-specific treatment, intravenous fluid replacement and antibiotics. 20 (80%) patients survived. Among survivors, 52% and 28% of isolates were Omicron BA.1 and BA.2, respectively, while patients who died had 16% Omicron BA.1 and 4% Omicron BA.2, respectively. There was no difference between Omicron BA.1 and BA.2 among survivors and those who died (*p* = 0.95) (Table 1).

**Table 1:** Demographic and clinical characteristics of transplant patients with confirmed omicron variant of COVID-19 by death status (29 Dec 2021–17 May 2022)

	Survived (*N* = 20)	Died (*N* = 5)	Total (*N* = 25)	*p* value*
Age (years)				
Mean ± SD	48.1 ± 14.4	54.4 ± 18.2	49.4 ± 15.0	0.433
≤ 50	9 (45.0%)	2 (40.0%)	11 (44.0%)	> 0.99
> 50	11 (55.0%)	3 (60.0%)	14 (56.0%)	
Gender				
Males	10 (50.0%)	4 (80.0%)	14 (56.0%)	0.341
Females	10 (50.0%)	1 (20.0%)	11 (44.0%)	
Comorbidity number				
Mean ± SD	2.2 ± 1.5	3.2 ± 1.8	2.4 ± 1.6	0.197
0–1	6 (30.0%)	1 (20.0%)	7 (28.0%)	> 0.99
2–3	11 (55.0%)	3 (60.0%)	14 (56.0%)	
≥ 4	3 (15.0%)	1 (20.0%)	4 (16.0%)	
Comorbidity				
Hypertension	7 (35.0%)	3 (60.0%)	10 (40.0%)	0.358
Diabetes	9 (45.0%)	4 (80.0%)	13 (52.0%)	0.322
Chronic kidney disease/ESRD	7 (35.0%)	3 (60.0%)	10 (40.0%)	0.358
Ischemic heart disease	1 (5.0%)	1 (20.0%)	2 (8.0%)	0.367
Cerebrovascular disease	2 (10.0%)	0 (0.0%)	2 (8.0%)	> 0.99
Chronic Lung disease	2 (10.0%)	0 (0.0%)	2 (8.0%)	> 0.99
Chronic liver disease/hepatitis	3 (15.0%)	1 (20.0%)	4 (16.0%)	> 0.99
Venous thromboembolism	2 (10.0%)	1 (20.0%)	3 (12.0%)	0.504
Others	10 (50.0%)	3 (60.0%)	13 (52.0%)	> 0.99
Transplant type				
Renal	14 (70.0%)	3 (60.0%)	17 (68.0%)	0.570
HPSCT	2 (10.0%)	1 (20.0%)	3 (12.0%)	
Lung	2 (10.0%)	0 (0.0%)	2 (8.0%)	
Liver	1 (5.0%)	1 (20.0%)	2 (8.0%)	
Heart	1 (5.0%)	0 (0.0%)	1 (4.0%)	
Immunosuppressive medications				
Mycophenolate	15 (75.0%)	5 (100.0%)	20 (80.0%)	0.544
Tacrolimus	18 (90.0%)	4 (80.0%)	22 (88.0%)	0.504
Steroids	16 (80.0%)	5 (100.0%)	21 (84.0%)	0.549
Others	4 (20.0%)	1 (20.0%)	5 (20.0%)	> 0.99
Omicron Subvariants				
Omicron BA.1	13 (52%)	4 (16%)	17 (68%)	0.95
Omicron BA.2	7 (28%)	1 (4%)	8 (32%)	
Hospitalization				
Home Isolation	2 (10.0%)	0 (0.0%)	2 (8.0%)	> 0.99
Hospitalization	18 (90.0%)	5 (100.0%)	23 (92.0%)	
COVID-19 specific treatment				
No	8 (40.0%)	0 (0.0%)	8 (32.0%)	0.140
Yes	12 (60.0%)	5 (100.0%)	17 (68.0%)	
COVID-19 specific treatment				
Steroid	8 (66.7%)	5 (100.0%)	13 (76.5%)	0.261
Remdesivir	6 (50.0%)	2 (40.0%)	8 (47.1%)	> 0.99
Tocilizumab	4 (33.3%)	3 (60.0%)	7 (41.2%)	0.593
Vaccination				
No	2 (10.0%)	1 (20.0%)	3 (12.0%)	0.504
Yes	18 (90.0%)	4 (80.0%)	22 (88.0%)	
Number of vaccination doses				
0	2 (10.0%)	1 (20.0%)	3 (12.0%)	0.717
1	1 (5.0%)	0 (0.0%)	1 (4.0%)	
2	9 (45.0%)	3 (60.0%)	12 (48.0%)	
3	8 (40.0%)	1 (20.0%)	9 (36.0%)	
Months between positive PCR and last vaccine dates				
Mean ± SD	4.3 ± 2.4	5.1 ± 5.2	4.4 ± 2.9	0.831
< 4	10 (55.6%)	2 (50.0%)	12 (54.5%)	> 0.99
≥ 4	8 (44.4%)	2 (50.0%)	10 (45.5%)	
Days between positive PCR and symptoms	5.2 ± 7.5	5.0 ± 4.8	5.1 ± 6.9	0.733
Severity of Illness				
Asymptomatic	2 (10.0%)	0 (0.0%)	2 (8.0%)	0.001
Upper respiratory tract infection	7 (35.0%)	0 (0.0%)	7 (28.0%)	
Pneumonia (not requiring oxygen)	5 (25.0%)	0 (0.0%)	5 (20.0%)	
Pneumonia (requiring low flow oxygen)	4 (20.0%)	0 (0.0%)	4 (16.0%)	
Pneumonia (requiring High flow oxygen)	1 (5.0%)	0 (0.0%)	1 (4.0%)	
Sever Pneumonia (requiring invasive ventilation)	1 (5.0%)	5 (100.0%)	6 (24.0%)	
ICU stay				
No	19 (95.0%)	0 (0.0%)	19 (76.0%)	< 0.001
Yes	1 (5.0%)	5 (100.0%)	6 (24.0%)	
Length of stay (mean ± SD days)				
ICU	8.0 ± NA	33.4 ± 27.1	29.2 ± 26.3	0.143
Hospital	6.8 ± 4.0	47.0 ± 31.1	15.6 ± 21.8	0.001

*Fisher exact test for categorical variables Mann Whitney test for continuous variables

6 (25%) patients had severe pneumonia and required invasive ventilation and intensive care unit (ICU) admission. Among ICU patients, 66.7% were more than 50 years, 50% had two to three comorbidities, 33% had three doses of vaccine, 83% had Omicron BA1, 5 out of 6 patients (83%) died (Table 2). Death was significantly associated with developing severe disease (*p* = 0.001) and ICU stay (*p* < 0.001).

**Table 2:** Demographic and clinical characteristics of transplant patients admitted to ICU with confirmed omicron variant of COVID-19 (29 Dec 2021–17 May 2022)

	ICU (N = 6)
Age (years)	
Mean ± SD	55.5 ± 16.5
≤ 50	2 (33.3%)
> 50	4 (66.7%)
Gender	
Males	4 (66.7%)
Females	2 (33.3%)
Comorbidity number	
Mean ± SD	3.5 ± 1.8
0–1	1 (16.7%)
2–3	3 (50.0%)
≥ 4	2 (33.3%)
Comorbidity	
Hypertension	4 (66.7%)
Diabetes	5 (83.3%)
Chronic kidney disease/ESRD	4 (66.7%)
Ischemic heart disease	1 (16.7%)
Cerebrovascular disease	0 (0.0%)
Chronic Lung disease	1 (16.7%)
Chronic liver disease/hepatitis	1 (16.7%)
Venous thromboembolism	1 (16.7%)
Others	4 (66.7%)
Transplant type	
Renal	4 (66.7%)
HPSCT	1 (16.7%)
Lung	0 (0.0%)
Liver	1 (16.7%)
Heart	0 (0.0%)
Immunosuppressive medications	
Mycophenolate	6 (100.0%)
Tacrolimus	5 (83.3%)
Steroids	6 (100.0%)
Others	1 (16.7%)
Omicron Subvariants	
Omicron BA.1	5 (83.3%)
Omicron BA.2	1 (16.7%)
COVID-19 specific treatment	
No	0 (0.0%)
Yes	6 (100.0%)
COVID-19 specific treatment	
Steroid	6 (100.0%)
Remdesivir	2 (33.3%)
Tocilizumab	4 (66.7%)
Vaccination	
No	1 (16.7%)
Yes	5 (83.3%)
Number of vaccination doses	
3 doses	2 (33.3%)
2 doses	3 (50.0%)
0 dose	1 (16.7%)
Months between positive PCR and last vaccine dates	
Mean ± SD	4.3 ± 4.8
< 4	3 (60.0%)
≥ 4	2 (40.0%)
Days between positive PCR and symptoms	5.0 ± 4.3
Severity of Illness	
Asymptomatic	0 (0.0%)
Upper respiratory tract infection	0 (0.0%)
Pneumonia (not requiring oxygen)	0 (0.0%)
Pneumonia (requiring low flow oxygen)	0 (0.0%)
Pneumonia (requiring High flow oxygen)	0 (0.0%)
Severe Pneumonia (requiring invasive ventilation)	6 (100.0%)
Length of stay (mean ± SD days)	
ICU	29.2 ± 26.3
Hospital	41.2 ± 31.3
Outcome	
Mortality	5(83.3%)

## Discussion

We described 20% mortality in a single-center cohort of hospitalized transplant patients in Saudi Arabia infected with Omicron variants while a recent study in USA showed that the mortality of hospitalized transplant recipients during the Omicron surge was only 4%. The main reason for the high mortality in our study is the lower three doses vaccination status compared to the other study; 36% versus 89% [6]. In addition, COVID-19 monoclonal antibodies were used in 74% of patients in the second study while none of our patients received sotrovimab. Another recent study in Spain showed that the mortality of Omicron variant was 16% in a cohort of hospitalized transplant recipients [7]. In this study, 93% of patients received three doses of COVID-19 vaccine status and they did not receive monoclonal antibodies indicating that three doses of vaccination were not enough to reduce the mortality of Omicron variants in patients with transplant recipients. Another recent study in Canada confirmed that the combination of three COVID-19 vaccine doses and early use of SARS-CoV-2 monoclonal antibodies were associated with prevention of progression to severe disease and oxygen requirements [8].

The main strength of our study is the inclusion of patients with confirmed Omicron based on whole genome sequence studies. We included patients with Omicron subvariant BA.1 and BA.2 and we did not show that specific Omicron subvariants were associated with poor outcome, recognizing that our study sample was not powered to demonstrate a difference in mortality between Omicron subvariants. There is no enough data so far to demonstrate that Omicron subvariants had different spectrum of severe diseases. A recent study in South Africa showed that Omicron B.A 1, 2, 4 and 5 surge is associated with reduced vaccine efficacy and increased hospitalization four months post booster vaccine and the authors suggested a booster vaccine four months post immunization or a new vaccine that included Omicron subvariants is required to reduce COVID-19 hospitalization [9].

The mortality in our study was observed in elderly patients with comorbidities, those who did not receive three doses of vaccine and required admission to the critical care unit. Age and comorbidities were the most important risk factors for mortality in transplant patients infected with COVID-19 in systemic reviews of several studies [10].

The reasons for the low uptake of booster COVID-19 vaccination among transplant recipients in our institution were not clear and deserve detailed evaluation. In our hospital, COVID-19 vaccine was available in the family medicine clinics without charges. Transplant patients were the main population target for COVID-19 booster vaccination according to our hospital guidelines. In addition, transplant recipients were counseled about the availability and efficacy of vaccination during their visits to the infectious diseases and nephrology clinics. We did not perform active surveillance among transplant recipients at different time periods to ensure that most patients were committed to receive the booster vaccination.

In our institution, there was a scarce supply of SARS-CoV-2 monoclonal antibodies. In addition, the use of monoclonal antibodies in transplant recipients was challenging during the various SARS-CoV-2 waves in view of their variable efficacy to various variants [11]. The use of tixagevimab-cilgavimab (Evusheld) was recommended as pre-exposure prophylaxis for COVID-19 in immunocompromised patients and it was associated with a reduction of SARS-CoV-2 incidence during the Alpha, Beta and Delta Surge. Subsequent in vitro studies showed that Evusheld was less effective in the prevention of COVID-19 in the presence of SARS-CoV-2 subvariants BA.4 and BA.5 and a high dose of Evusheld was recommended based on pharmacodynamics and pharmacokinetics data [12]. Recently, a new study confirmed that the neutralizing activity of Evusheld was inefficient against the new subvariants SARS-CoV-2 BQ and XBB and its use should be carefully evaluated when these subvariants were predominant in the community [13]_._

According to our hospital protocol and based on recent evidence favoring the early use of remdesivir in immunocompromised patients to prevent progression to severe disease, transplant patients presenting with mild to moderate disease in our institution were eligible to receive three days of intravenous remdesivir [14]. There was no difference in the utilization of remdesivir between survivors and non-survivors, though our study was not powered to evaluate the impact of remdesivir on mortality.

Our study had several limitations. We did not include non-hospitalized patients that tend to have mild disease and better outcome. The overall mortality would be lower in case our study population included non-hospitalized patients. We included a heterogenous population of solid organ transplant and hematopoietic stem cell transplant recipients with variable immunosuppressive status which could over-estimate the overall mortality. We did not perform SARS-CoV-2 serological tests in both survivors and non-survivors which could be a predictor of mortality in our study population in view of the low percentage of patients receiving a third booster vaccine dose.

## Conclusions

In our single-center study of transplant recipients infected with Omicron, the mortality rate was high in patients who required critical care admission. Only one third of transplant patients received a third booster COVID-19 vaccine. The use of booster vaccine dose is a fundamental COVID-19 preventive component in any transplant centers. The use of monoclonal antibodies was challenging in immunocompromised patients during COVID-19 pandemic as various medications were losing their neutralizing antibodies in the presence of emerging SARS-CoV-2 subvariants that had different mutations and immune escape mechanisms. There was no difference between Omicron subvariants B.A.1 and B.A.2 in survivors and those who died though our study was not powered for this objective. Future studies are required to understand the impact of Omicron subvariants on the outcome of immunocompromised patients.

## Data Availability

Data and materials are available when requested.

## Abbreviations

ESRD: End stage renal disease
Evusheld: Tixagevimab–cilgavimab
ICU: Intensive care unit
KAUST: King Abdullah University of Science and Technology
KFSHRC-R: King Faisal Specialist Hospital and Research Center, Riyadh
KFSHRC-J: King Faisal Specialist Hospital and Research Center, Jeddah
PCR: Polymerase chain reaction
SD: Standard deviation
USA: United States of America
